# Peritoneal mesothelioma in a woman who has survived for seven years: a case report

**DOI:** 10.1186/1752-1947-5-36

**Published:** 2011-01-26

**Authors:** Krishna Pillai, Javed Akhter, Mohammad H Pourgholami, David L Morris

**Affiliations:** 1 Department of Surgery, St. George Hospital, University of New South Wales, Kogarah, NSW, Australia

## Abstract

**Introduction:**

Malignant peritoneal mesothelioma is a rare cancer with poor patient survival. Female gender has been identified as a positive prognostic factor. Recently, it has been suggested that the expression of estrogen receptor β in malignant mesothelioma leads to tumor suppression and a better prognosis.

**Case presentation:**

We report the case of a 48-year-old Caucasian woman who is alive and disease-free seven years after the initial diagnosis and treatment of malignant peritoneal mesothelioma.

**Conclusion:**

This patient's long survival may be attributable to a combination of factors, including minimal disease, complete cytoreductive surgery and hyperthermic intraperitoneal chemotherapy plus the estrogen receptor β positivity of the tumor.

## Introduction

Peritoneal mesothelioma is a rare but fatal disease; the incidence is approximately one per million, and peritoneal mesothelioma accounts for about 20% to 30% of all cases of mesothelioma [1]. Although asbestos has been implicated as the main carcinogen [2,3], other factors such as radiation, peritonitis and SV40 have all been implicated [4].

Peritoneal mesothelioma progresses with unspecific symptoms, and when presented, it is commonly in the form of increased abdominal girth, pain and weight loss; hence, diagnosis is late, with a poor prognosis. A number of therapeutic regimens have been used to improve prognosis [5], and currently debulking surgery is followed by hyperthermic intraperitoneal chemotherapy (HIPEC). This has led to marked improvement in patients who were once classified as preterminal [6]. The current median survival is around 10 months, and relative five-year survival is in approximately 16% [7]. Hence, more information on the disease and more effective therapies are needed to improve prognosis.

## Case presentation

A Caucasian women, now aged 48 years, presented herself at the age of 40 years with abdominal pain (four to five days), a bad taste in her mouth and tiredness. She had epigastric discomfort caused by abdominal distension for the past four to five years and had multiple upper and lower gastrointestinal endoscopic examinations. Her medical history involved obesity, treatment for a blocked salivary duct, hypertension, endometriosis, appendectomy, Bell's palsy and hormone replacement therapy. Recent laproscopic cholecystectomy showed areas of abnormality, and a biopsy revealed the presence of malignant mesothelioma of epithelial histology. A computed tomography (CT) scan showed peritoneal tumor, not widely spread with no parenchymal liver disease. There were no pleural nodules or fluid collection along with the absence of metastasis. Disease volume as determined by peritoneal cancer index was low. The patient denied any exposure to asbestos.

Tumor markers such as CA125 appeared to be normal and ranged from 7 to 11, which fell within the reference range (0-35 IU/mL). Blood analysis revealed that the patient had mild to moderate anemia with moderate thrombocytosis.

Laparotomy with peritonectomy performed one month later revealed the accumulation of ascitic fluid (four litres) with no liver disease but some disease affecting the diaphragm, small bowel, colon and uterus. Complete cytoreduction was carried out, with preservation of the spleen (minor diseased part removed). Similarly, disease affecting the small bowel, mesentery and colon was also removed. Disease in the uterus was diathermised, and HIPEC was carried out with 50 mg/m^2 ^of cisplatin and 15 mg/m^2 ^of Adriamycin for 90 minutes at 41.5°C and 20 mg/m^2 ^(5 cycles) of paclitaxel, with insertion of a peritoneal catheter and port. The patient refused postoperative chemotherapy.

Macroscopic findings showed a peritoneal tumor (multiple pieces of omentum 400 × 200 × 50 mm in aggregates), and microscopic investigation showed some areas of prominent papillary tumor on mesothelial surface. Nuclear atypia varied from minimal to focally moderate with nuclear membrane irregularities and anisonucleosis. Mitosis was rare (<1/10 high-power field). Further focal stromal invasion of small groups of cells and single cells was seen in underlying fat with an absence of desmoplastic response. Very rare psammoma bodies were seen, and necrosis was absent in this section. Atypical mesothelial proliferation was also seen in all sections examined. Chronic inflammation was also seen in the subserosal connective tissues. Although the tumor was WDPM entering into differential diagnosis, the extent of the disease and the presence of invasion mitigated against this diagnosis. The immunohistochemical (ICH) findings are shown in Table 1.

**Table 1:** Protein markers that have been identified by immuno histochemistry in the patient tumor samples

Protein Markers	Positive (+)	Negative (-)
CAM5.2	+	

Cytokeratin	+	

HBME-1	+ (thick or membranous)	

CD 15		-

BER-EPA		-

CEA		-

Human epithelial antigen		-

EMA	+ (focal staining)	

Two years later, the patient presented herself with epigastric discomfort, gastric reflux, abdominal pain, constipation and diarrhea. A CT scan was normal, but the patient underwent laparotomy and a second peritonectomy. The findings were adhesions (significant at terminal small bowel and right colon), few nodules (bowels and mesentery) and a thin membranous septum on the small bowel. Macroscopic examination showed occasional atypical cells with minor peritoneal disease (epitheloid cells) consistent with mesothelioma. There was absence of tumor in lymph nodes, the lesser omentum and the hepatic artery. Microscopic examination results are shown in Table 2. Hence, it was concluded that very low-volume disease was present, and pain was mainly attributable to adhesions.

**Table 2:** Diagnostic findings from patient specimens (formalin fixed and paraffin embedded)

Section	Result of Examination
Formalin fixed	Atypical epithelioid cells present
	Higher nuclear:cytoplasmic ratio

Paraffin embedded	Presence of nodules of cellular tumor
	Positive for HBME-1 and EMA
	Absence of tumor in lymph nodes
	Adipose tissue, omentum: low-grade epithelioid mesothelioma

Treatment with HIPEC (cisplatin 200 mg + mitomycin C 25 mg/90 min/41.5°C), extensive division of adhesions, peritoneal biopsy and intraoperative ultrasonography were carried out. The membranous septum on the small bowel along with two nodules were also removed. The patient refused to have postoperative chemotherapy.

Seven months later, the patient presented with a paraspinal mass, and microscopic examination showed no tumor; the patient was negative for CK5/6, cytokeratin 5.2, epithelial membrane antigen and mesothelial cell membrane protein. Therefore, diagnosis was made in favor of fibrosis and mild chronic inflammation. Another 11 months later, the patient underwent laparotomy during which division of adhesions and repair of a hernia was carried out. No evidence of mesothelioma was found at this stage, and two months later, laparotomy was repeated for division of adhesions and debridement of a large abscess owing to infection. The wound did not heal, so the patient was prescribed antibiotic therapy with an open wound-healing regimen. Finally, the wound resolved, and currently the patient is well.

## Discussion

Although the tumor was WDPM entering into differential diagnosis, the extent of the disease and the presence of invasion mitigated against this diagnosis. Hence, the patient underwent cytoreductive surgery with HIPEC, which is most effective for patients with malignant peritoneal mesothelioma (MPM). Only two peritonectomies with HIPEC were carried out in this patient, and she did not receive postoperative chemotherapy. Although the patient developed some complications as a result of adhesions, this was rectified during recovery with subsequent laparotomy. CT scans and ICH findings showed the absence of any malignancy after the second HIPEC.

Unlike many patients, who succumb to the disease soon after treatment, this patient is alive and well past seven years. Close scrutiny of the case seems to reveal three salient features that may have contributed to this person's favorable prognosis. First, it appears from diagnosis (macroscopic and microscopic) that the patient may have a less aggressive form of MPM (low disease volume with no metastasis) that with complete cytoreduction is probably more amenable to HIPEC. This conclusion has been derived because the patient responded well to HIPEC treatment in the first three years, after which her examination revealed abatement of the disease. Indeed, the last laparotomy performed did not reveal any disease.

Second, the pathology report indicated that the patient had an epithelioid type of MPM with abundant cellular cytoplasm that is less aggressive and more amenable to HIPEC compared with the sarcomatous or mixed type [8].

**Figure 1:**
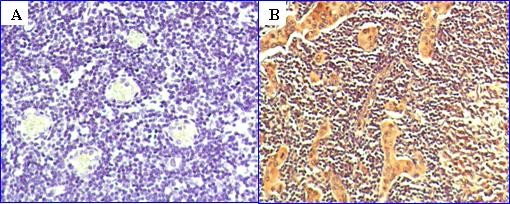
**Immunohistochemical staining of paraffin-embedded slides (3 μm in thickness)**. **A - **Negative for estrogen receptor β (ER_β_) (breast tissue) stained blue. **B - **Patient slide with heavy staining for ER_β, _stained brown.

Finally, immunohistochemical examination of the tumor tissues revealed that the patient has a high expression of estrogen receptor β (ER_β_) (Figure 1). Very recent studies by Pinton *et al. *[9] have indicated that ER_β _expression in pleural mesothelioma has prognostic significance and that high expression of these receptors has endowed marked longevity in these patients. These authors have also suggested that manipulation of ER_β _receptors may offer a new mode of therapy for this type of cancer. Our studies have also shown that the expression of ER_β _in peritoneal mesothelioma offers a better prognosis (unpublished data).

Noticeably, this patient's plasma estradiol was measured to be 483 ρmol/L, which is comparable to levels found in women during the follicular phase of the ovarian cycle. Estradiol is a universal ligand for both ER_α _and ER_β_. ER_β _is endowed with modulatory function on ER_α_-dependant cell proliferation [10], and when present by itself, it is able to control cell replication through the G2-M phase cell arrest in a ligand-dependant and -independent manner [9]. Hence, it may be suggested that the high level of estradiol together with the high expression of ER_β _could have led to better disease control and hence longer survival.

## Conclusion

Taken as whole, cytoreduction with HIPEC has conferred good prognosis on this patient owing to the mild nature of the disease of epitheloid histology with ER_β _expression and high plasma estradiol level.

## Abbreviations

CT: computed tomography; ER_β_: estrogen receptor β; HIPEC: hyperthermic intraperitoneal chemotherapy; ICH: immunohistochemical; MPM: malignant peritoneal mesothelioma; WDPM well-differentiated papillary mesothelioma.

## Competing interests

The authors declare that they have no competing interests.

## Consent

Written informed consent was obtained from the patient for publication of this case report and any accompanying images. A copy of the written consent is available for review from the Editor-in Chief of the journal.

## Authors' contributions

KP, MHP, JA and DLM collected, analyzed and interpreted patient data. KP was the major contributor in writing the manuscript. All authors read and approved the final manuscript.
